# Supplementary material for: EnzML: multi-label prediction of enzyme classes using InterPro signatures
Source: BMC Bioinformatics. 2012 Apr 25;13:61. doi: 10.1186/1471-2105-13-61 (PMC3483700; doi:10.1186/1471-2105-13-61)
Supplement: Addtional file 5 — The Java code to format the data files, evaluate and predict. The file enzml_java_code.tar.gz contains the Java code used to format database data to ARFF and XML formats, to execute cross and train-test (jackknife) evaluations and to record evaluation results to database. More information is included in the readme.txt file and the Javadoc files. The code can be used with a MySQL database. To use a different database software, other JDBC drivers might be required. [file 1471-2105-13-61-S5.gz › java_code/enzml2011/doc/uk/ac/ed/inf/csb/taweka/test/PathTest.html]

PathTest


---


|  |  |  |
| --- | --- | --- |
| **Overview** \| **Package** \| **Class** \| **Use** \| **Tree** \| **Deprecated** \| **Index** \| **Help** | |  |
| **PREV CLASS**   **NEXT CLASS** | **FRAMES**    **NO FRAMES**     **All Classes** |  |
| SUMMARY: NESTED \| FIELD \| CONSTR \| METHOD | DETAIL: FIELD \| CONSTR \| METHOD |  |


---


## uk.ac.ed.inf.csb.taweka.test Class PathTest

```
java.lang.Object
  uk.ac.ed.inf.csb.taweka.test.PathTest
```

---

``` public class PathTest extends java.lang.Object ```

Contains file paths for test (database properties files etc.)

**Version:**
:   14 Jun 2010

**Author:**
:   Luna De Ferrari luna.deferrari-at-ed.ac.uk

---

| **Field Summary** | |
| --- | --- |
| `static java.lang.String` | `ARCHAEA_XML` |
| `static java.lang.String` | `BACTERIA_XML` |
| `static java.lang.String` | `DATA_PROPERTIES_SWISS_KEGG_100` |
| `static java.lang.String` | `DATA_PROPERTIES_SWISS_KEGG_1000` |
| `static java.lang.String` | `DATA_PROPERTIES_SWISS_KEGG_10000` |
| `static java.lang.String` | `DATA_PROPERTIES_SWISS_KEGG_ARCHAEA` |
| `static java.lang.String` | `DATA_PROPERTIES_SWISS_KEGG_BACTERIA` |
| `static java.lang.String` | `DATA_PROPERTIES_SWISS_KEGG_FUNGI` |
| `static java.lang.String` | `DATA_PROPERTIES_SWISS_KEGG_PLANTS` |
| `static java.lang.String` | `DATA_PROPERTIES_SWISS_KEGG_VERTEBRATES` |
| `static java.lang.String` | `DATA_PROPS_SWISS_KEGG` |
| `static java.lang.String` | `FUNGI_XML` |
| `static java.lang.String` | `MACHINE_LEARNING_PROPERTIES`             Machine learning properties: contains the database to save the Mulan evaluation results to. |
| `static java.lang.String` | `MULAN_XML_UNIPROT` |
| `static java.lang.String` | `MULAN_XML_UNIPROT_RANDOM_100` |
| `static java.lang.String` | `MULAN_XML_UNIPROT_RANDOM_1000` |
| `static java.lang.String` | `MULAN_XML_UNIPROT_RANDOM_10000` |
| `static java.lang.String` | `PLANTS_XML` |
| `static java.lang.String` | `TEST_DATA_PROPERTIES`             Taweka data properties: contains database connection properties and data queries to generate a Weka/Mulan ARFF file |
| `static java.lang.String` | `TEST_DB_CONN_PROPERTIES`             Contains database connection properties |
| `static java.lang.String` | `TEST_MACHINE_LEARNING_PROPERTIES`             Machine learning properties: contains the database to save the Mulan evaluation results to. |
| `static java.lang.String` | `TEST_RESULTS_FOLDER` |
| `static java.lang.String` | `VERTEBRATES_XML` |


**Constructor Summary**

`PathTest()`


**Method Summary**

**Methods inherited from class java.lang.Object**

`equals, getClass, hashCode, notify, notifyAll, toString, wait, wait, wait`

**Field Detail**

### ARCHAEA\_XML

```
public static final java.lang.String ARCHAEA_XML
```

**See Also:**: Constant Field Values

---


### BACTERIA\_XML

```
public static final java.lang.String BACTERIA_XML
```

**See Also:**: Constant Field Values

---


### DATA\_PROPERTIES\_SWISS\_KEGG\_100

```
public static final java.lang.String DATA_PROPERTIES_SWISS_KEGG_100
```

**See Also:**: Constant Field Values

---


### DATA\_PROPERTIES\_SWISS\_KEGG\_1000

```
public static final java.lang.String DATA_PROPERTIES_SWISS_KEGG_1000
```

**See Also:**: Constant Field Values

---


### DATA\_PROPERTIES\_SWISS\_KEGG\_10000

```
public static final java.lang.String DATA_PROPERTIES_SWISS_KEGG_10000
```

**See Also:**: Constant Field Values

---


### DATA\_PROPERTIES\_SWISS\_KEGG\_ARCHAEA

```
public static final java.lang.String DATA_PROPERTIES_SWISS_KEGG_ARCHAEA
```

**See Also:**: Constant Field Values

---


### DATA\_PROPERTIES\_SWISS\_KEGG\_BACTERIA

```
public static final java.lang.String DATA_PROPERTIES_SWISS_KEGG_BACTERIA
```

**See Also:**: Constant Field Values

---


### DATA\_PROPERTIES\_SWISS\_KEGG\_FUNGI

```
public static final java.lang.String DATA_PROPERTIES_SWISS_KEGG_FUNGI
```

**See Also:**: Constant Field Values

---


### DATA\_PROPERTIES\_SWISS\_KEGG\_PLANTS

```
public static final java.lang.String DATA_PROPERTIES_SWISS_KEGG_PLANTS
```

**See Also:**: Constant Field Values

---


### DATA\_PROPERTIES\_SWISS\_KEGG\_VERTEBRATES

```
public static final java.lang.String DATA_PROPERTIES_SWISS_KEGG_VERTEBRATES
```

**See Also:**: Constant Field Values

---


### DATA\_PROPS\_SWISS\_KEGG

```
public static final java.lang.String DATA_PROPS_SWISS_KEGG
```

**See Also:**: Constant Field Values

---


### FUNGI\_XML

```
public static final java.lang.String FUNGI_XML
```

**See Also:**: Constant Field Values

---


### MACHINE\_LEARNING\_PROPERTIES

```
public static final java.lang.String MACHINE_LEARNING_PROPERTIES
```

:   Machine learning properties: contains the database to save the Mulan
    evaluation results to.

    **See Also:**: Constant Field Values

---


### MULAN\_XML\_UNIPROT

```
public static java.lang.String MULAN_XML_UNIPROT
```

---


### MULAN\_XML\_UNIPROT\_RANDOM\_100

```
public static java.lang.String MULAN_XML_UNIPROT_RANDOM_100
```

---


### MULAN\_XML\_UNIPROT\_RANDOM\_1000

```
public static final java.lang.String MULAN_XML_UNIPROT_RANDOM_1000
```

**See Also:**: Constant Field Values

---


### MULAN\_XML\_UNIPROT\_RANDOM\_10000

```
public static java.lang.String MULAN_XML_UNIPROT_RANDOM_10000
```

---


### PLANTS\_XML

```
public static final java.lang.String PLANTS_XML
```

**See Also:**: Constant Field Values

---


### TEST\_DATA\_PROPERTIES

```
public static final java.lang.String TEST_DATA_PROPERTIES
```

:   Taweka data properties: contains database connection properties and data
    queries to generate a Weka/Mulan ARFF file

    **See Also:**: Constant Field Values

---


### TEST\_DB\_CONN\_PROPERTIES

```
public static final java.lang.String TEST_DB_CONN_PROPERTIES
```

:   Contains database connection properties

    **See Also:**: Constant Field Values

---


### TEST\_MACHINE\_LEARNING\_PROPERTIES

```
public static final java.lang.String TEST_MACHINE_LEARNING_PROPERTIES
```

:   Machine learning properties: contains the database to save the Mulan
    evaluation results to.

    **See Also:**: Constant Field Values

---


### TEST\_RESULTS\_FOLDER

```
public static final java.lang.String TEST_RESULTS_FOLDER
```

**See Also:**: Constant Field Values

---


### VERTEBRATES\_XML

```
public static final java.lang.String VERTEBRATES_XML
```

**See Also:**: Constant Field Values


**Constructor Detail**

### PathTest

```
public PathTest()
```


---


|  |  |  |
| --- | --- | --- |
| **Overview** \| **Package** \| **Class** \| **Use** \| **Tree** \| **Deprecated** \| **Index** \| **Help** | |  |
| **PREV CLASS**   **NEXT CLASS** | **FRAMES**    **NO FRAMES**     **All Classes** |  |
| SUMMARY: NESTED \| FIELD \| CONSTR \| METHOD | DETAIL: FIELD \| CONSTR \| METHOD |  |


---
